# Whole-genome sequence and annotation of *Diaporthe australafricana* strain CBS 118571

**DOI:** 10.1128/mra.00515-24

**Published:** 2024-08-30

**Authors:** Xin Li, Xinyi Wang, Limei Li, Rong Lei

**Affiliations:** 1 Technology Center of Dalian Customs District, Dalian, China; 2 School of Life and Health, Dalian University, Dalian, China; 3 Jilin Provincial Academy of Forestry Science, Changchun, China; 4 Chinese Academy of Inspection and Quarantine, Beijing, China; University of Maryland School of Medicine, Baltimore, Maryland, USA

**Keywords:** whole-genome sequence, annotation, *Diaporthe australafricana*

## Abstract

We report the whole-genome sequence of *Diaporthe australafricana* Crous & J.M. van Niekerkusing using Oxford Nanopore long-read sequencing and Illumina short-read sequencing. The hybrid genome consists of 11 contigs with a total length of 53.509 Mb, and a GC content of 52.40%.

## ANNOUNCEMENT

*Diaporthe australafricana* Crous & J.M. van Niekerkusing strain CBS 135771 was originally isolated from grapevines in the Western Cape province, Stellenbosch in South Africa (1), and deposited at CBS-KNAW, Utrecht, Netherlands. *D. australafricana* is highly virulent in shoots, stems, and fruits of blueberry (2), causing stem canker and dieback, lesions on stems, and necrosis of shoots (3). Here, we performed *de novo* assembly and annotation of *D. australafricana* genome using Oxford Nanopore long-read sequencing and Illumina short-read sequencing.

The white and fluffy aerial mycelium colonies were cultivated on a PDA culture medium, and grown at 20°C under a 12-h dark/12-h light cycle for approximately 6 days. The mycelia were collected and ground with liquid nitrogen, then the genomic DNA was extracted using a DNeasy Plant Pro Kit (Qiagen, GmbH, Hilden, Germany), and the total RNA was extracted using an RNAprep Pure Plant Plus Kit (Tiangen, Beijing, China). The RNA integrity number was analyzed with an Agilent RNA 6000 Nano Kit on an Agilent 2100 Bioanalyzer (Agilent, Santa Clara, CA, USA). To construct the ONT DNA library, genomic DNA was fragmented with a g-Tube (Cat No. 520079, Covaris, USA) and the long DNA fragments were retained with SPRlselect Reagent (Cat No. B23318, Beckman, USA). Next, NEBNext FFPE Repair Mix (Cat# M6630) and NEBNext Ultra II End Repair/dA-tailing Kit (Cat# E7546) were used to end-repair and A-tailing for the fragmented DNA. The adapter in the SQK-LSK109 (ONT, UK) was mixed with NEBNext Quick Ligation Reaction Buffer (Cat# B6058) and NEB T4 DNA Ligase 2 M U/mL (Cat# M0202) for ligation. Approximately 600 ng library was loaded to a MinION Flow Cell (R9.4.1) in an Oxford Nanopore promethION P48 platform (Oxford Nanopore Technologies, UK) for sequencing. For Illumina sequencing, 1 µg genomic DNA was fragmented using NEBNext dsDNA Fragmentase (Cat# M0348), and library was constructed using the ExCell Bio Library Construction Kit (NuGEN, San Carlos, CA, USA). Illumina RNA-Seq library was constructed with the TransNGS Stranded RNA-Seq Library Prep Kit (TransGen, Beijing, China). The quality control of libraries was performed on an Agilent 2100 Bioanalyzer, and the libraries were sequenced on an Illumina NovaSeq 6000 platform (San Diego, CA, USA). For Nanopore sequencing, the basecalling from the raw FAST5 files was performed using guppy (v6.5.7) (4) with the basecalling model of dna_r9.4.1_450bps_hac, followed by adapter trimming using PoreChop (v0.2.4) (5). For Illumina sequencing, the raw data (12,453,433,200 bp with PE150 and 893,925,500 bp with PE250) were trimmed of adapters and low-quality reads (mean quality value <20) by using skewer (6) (v0.2.2). Default parameters were used for the software except where otherwise noted.

The hybrid assembly of Illumina data sets and Nanopore long reads into contigs was performed using MaSuRCA (7) (v3.3.7). The completely assembled *D. australafricana* CBS 135771 contained 11 contigs with a cumulative size of 53.509 Mb and an *N*_50_ length of 5.75 Mb. The concordance and the integrity of the assembly were determined using Burrows-Wheeler Alignment tool (BWA, version: 0.7.13-r1126) (8) and BUSCO (9, 10) (v4.1.4) , respectively. and the genome mapping ratios were 99.56% and 99.07%, respectively. All metrics regarding assembly are listed in Table 1.

**TABLE 1:** Genome feature of *D. australafricana* CBS 135771

Features	Strain CBS135771
ONT long reads (sequencing depth)	7.748 Gb (~144.8×)
Average read length	18,252.08
Reads *N*_50_	26,242
Maximum read length	135,101
Illumina short reads (sequencing depth)	13.35 Gb (~249.4×)
Estimated genome size	52.90 Mb
Estimated genome repeats	6.06%
Estimated genome heterozygosity	0.08%
RNA-Seq	6.31 Gb (~117.9×)
Assemble size	53.51 Mb
Contig number	11
Contig *N*_50_	5,751,947
Contig *L*_50_	4
Average contig length	4,864,461.45
Maximum contig length	11,543,331
GC content	52.40%
Repeat sequence	6.60%
BUSCO fungal integrity	99.0%
Illumina reads mapping rate	99.56%
ONT long reads mapping rate	99.07%

The assembled genome was annotated using pipeline named funannotate (11) (v1.8.3), in which a genome-guided assembly process of RNA-Seq reads by Trinity (12) was invoked. Finally, genome annotation predicted annotated 15,235 protein-coding genes.

## Data Availability

The whole-genome data of D. australafricana CBS 135771 presented here have been deposited at GenBank under the accession number JBCEDM000000000. The accession numbers of BioProject and BioSample are PRJNA1091203, and SAMN 40589631, respectively. The SRA accession number of ONT reads, Illumina PE150 reads, Illumina PE250 reads, and RNA-Seq PE150 reads are SRR29120074, SRR29120077, SRR29120076 and SRR29120075, respectively.
